# ‘It’s a secret in my life’: frontstage and backstage experiences of women undergoing induced abortions in a rural community of Pakistan

**DOI:** 10.1186/s12905-024-03482-5

**Published:** 2024-12-31

**Authors:** Rubeena Slamat, Piet Bracke, Melissa Ceuterick

**Affiliations:** 1 Department of Sociology, Faculty of Political & Social Sciences, Ghent University, Ghent, Belgium; 2 Department of Sociology, Ghent University, Campus-UFO, Technicum T 1, Sint-Pietersnieuwstraat 41, Ghent, B-9000 Belgium

**Keywords:** Abortion, Stigma management, Frontstage-backstage, Punjab-Pakistan

## Abstract

Abortion is taboo in Pakistani society, and it is stigmatised throughout the country, regardless of legal permission. Despite the burdens imposed by social norms of pronatalism and motherhood, women do exercise agency to have abortions. This study explores the experiences of women who have had induced abortions and maintained their social status within the community. Goffman’s dramaturgical theory (frontstage and backstage behaviours) is applied in order to document these experiences. Data were collected through 38 in-depth interviews and six focus group discussions. The results are presented according to a grounded theory approach. The results of this study indicate that women exercise agency and stage abortions as miscarriages, which are culturally more acceptable. They do this with the help of support systems comprising family members and friends. They are able to maintain their social status by navigating between what Goffman refers to as the frontstage and backstage regions of their lives. In this study, these regions are not defined by physical boundaries, but by the presence of participants and audience. Women must therefore take care to behave appropriately, especially in the frontstage region. Even when women do opt for abortion, the experience is likely to result in the psychological burden of a lifelong memory.

**Supplementary Information:**

The online version contains supplementary material available at 10.1186/s12905-024-03482-5.

## Introduction

### Abortion in Pakistan

Medical professionals define induced abortion as the termination of a pregnancy before the foetus has attained viability, become capable of independent extra-uterine life [1, 2]. Abortions occur worldwide, and it has been estimated that one in every five pregnancies ends in abortion globally [3]. Abortion rates (i.e. the number of abortions per 1,000 women between the ages of 15 and 44 years) vary from region to region. Studies have established the abortion rate in Pakistan at 29 per 1,000 women [4, 5], and the Guttmacher Institute reports that two million unsafe abortions occur in Pakistan each year [6, 7]. The fertility rate in Pakistan is 3.47 children per woman [8, 9]. According to the Guttmacher Institute, 9,720,000 pregnancies occurred each year in Pakistan between 2015 and 2019. Of these pregnancies, 3,690,000 were unintended, and 2,240,000 ended in abortion [10]. In addition to cross-national variations in abortion rates, each country has its own legal sanctions for abortions, and these permissions and restrictions also have an impact on the abortion rate. Factors that affect abortion rates thus include the laws, cultural norms and values of specific countries.

The objective of this study is to document the experiences of women who have undergone induced abortion within the patriarchal,^1^ pronatalist^2^ society of Pakistan, where abortion is taboo. In Pakistan, abortion is viewed negatively for a myriad of reasons, including legal and religious considerations. Pakistani abortion legislation has undergone several amendments since the early 1990s. Prior to 1990, abortion was criminalised under the Pakistan Penal Code, unless it was performed in ‘good faith to save the woman’s life’. This provision was meant to ensure conformity with the injunctions of Islam, in line with the guidance in the Holy Quran and Sunnah. A 1997 amendment of the Penal Code expanded legal permission to perform abortions in the early stages of pregnancy (16 weeks) beyond ‘saving the life of the woman’ to include ‘necessary treatment’ [11]. At present, therefore, legal permission (in-line with religious code) for abortion in Pakistan depends on gestational age, and it is legally allowed to save the woman’s life or to provide necessary treatment before foetal organs have formed. After the foetal organs have formed, abortion is allowed only to save the woman’s life [12].

In addition to gestational considerations, religious authorities argue that permissions and restrictions relating to abortion depend upon ensoulment. According to Islamic beliefs, ensoulment occurs in the fourth month of gestation, after which the foetus is regarded as a human being. Following Islamic religious argumentation, therefore, abortion is allowable before the fourth month of pregnancy [13]. Legal permissions operate primarily at the hospital level, where practitioners discourage abortion if there is no medical reason that poses a threat to the woman and/or the foetus. Moreover, hospital practitioners may refuse to assist in abortion, citing religious reasons. Studies have indicated that trained healthcare providers often demonstrate negative attitudes towards women seeking abortion, due to conflicting personal and professional values, as well as to their limited knowledge and skills regarding the safe abortion methods recommended by the WHO [14]. In practice, therefore, the prevailing legal and religious permissions in Pakistan do not operate as effectively as desired [15, 16]. As a result, most women who choose to have abortions are likely to do so through self-medication using abortive home remedies, after which they present in hospital with supposed symptoms of miscarriage. Alternatively, they may opt to undergo clandestine abortion with the help of local service providers (e.g. traditional birth attendants or ‘TBAs’) [5, 10].

From an international perspective, the rate of miscarriage in Pakistan (13% of all pregnancies) [17] is relatively high. According to estimates by Sathar, only one out of five women visit a hospital after an abortion [5]. Another reason for clandestine abortion is the price of service providers [10]. In Pakistan, the cost of abortion depends upon the type of service provider, as the fees charged by professional doctors are higher than those charged by local service providers (TBAs) [10]. The cost of an abortion is at least USD 20, which is a considerable amount, given the average monthly income is only USD 138.66. The per capita annual income in Pakistan is USD 1,663.99 [18].

Aside from the cost, the most common reason for deciding to have a clandestine abortion is the social stigma associated with abortion within the prevailing norms of Pakistan [10]. The logic underlying the negative social perception of abortion is that it is contrary to the norms of motherhood and pronatalism. In the pronatalist society of Pakistan, abortion is strongly contested and highly unacceptable. Children are regarded as the cornerstone of the community and a necessity for perpetuating the lineage and family. This social value makes children beneficial to society, families and couples, and especially to women. This is because, in Pakistani society, ‘motherhood’ is the status required of every married woman [19–21]. Motherhood allows women to enjoy an array of status and powers associated with it. Stated simply, a child constitutes a woman’s identity. The status of ‘mother’ is always accompanied by responsibilities. As argued by Ruddick [22], motherhood entails three responsibilities: to preserve the child’s life, to foster the child’s growth and to do this according to the values of the group. As a mother, a woman is expected to maintain a focus on the child’s benefit and take care of the child in all circumstances, good or bad. Women are not considered capable of harming their children [23]. Under this norm, women alter their lifestyles to the child’s benefit from the time at which pregnancy is confirmed. In Pakistani society, therefore, abortion is surrounded by legal, religious and social forces. Furthermore, as observed in some studies, most Pakistani people are unaware of the prevailing laws on abortion [24], either legal or religious. Abortion is thus not socially accepted within the Pakistani communities, and women who opt for abortion are subject to stigma [25], as is the case in many parts of the world, regardless of laws and permissions [26–29]. Similarly, many women in Pakistan secretly opt for abortion, as documented in Chahal’s study on medication-induced abortions [25] and Sultana’s study on traditional abortion methods [30]. Both studies reveal that despite societal expectations, some women make private decisions to terminate pregnancies. The present study focuses on the experiences of women who have undergone abortion but maintained their social status within a society that stigmatises abortion.

### Abortion stigma

Goffman [31] defines stigma as an attribute that is highly discrediting—a ‘mark’ or ‘label’ that characterises a person as ‘deviant’ in the eyes of society. In the theory of stigma, Goffman (p. 4) identifies three types of stigma: (1) ‘abominations of the body’, which refer to physical deformities (e.g. disabilities) that result in an appearance that is not in accordance with normative cultural standards; (2) ‘blemishes of individual character’, which refer to weak will and moral characteristics that are contrary to dominant cultural norms (e.g. mental disorders, homosexuality and induced abortion); and (3) ‘tribal stigma’, which applies to members of a group, nation or race and are perpetuated from one generation to another [31]. Abortion stigma falls within the second type of stigma. Such marking devalues individuals in social interactions and has an impact on their identity. As defined by Kumar and colleagues [32], abortion stigma is a negative attribute ascribed to women who seek to terminate a pregnancy that marks them—either internally or externally—as inferior to the ideal of ‘womanhood’. Proceeding from the stigma framework developed by Link and Phelan [33], which holds that stigma is exercised due to an imbalance of power within society, Kumar discusses how negative stereotypes are used to distinguish and label these women, who then face isolation and discrimination in everyday life. Abortion stigma falls under two of the three types of stigma outlined by Goffman [31]: blemishes of character and tribal stigma. Abortion is seen as a sin that blemishes a woman’s moral character and that runs counter to the norms of femininity and motherhood. As argued by Nack [34], gendered norms of sexual morality have shaped at least two distinct ‘tribes’ of women: the tribe of ‘good’ girls/wives/mothers enjoys a higher status than does the tribe of ‘bad girls and fallen women’. The latter tribe consists of women who have been socially devalued for defying social expectations of feminine ‘goodness’, especially with regard to sexuality and motherhood. The behaviours that trigger and surround the bad tribe are perceived as individual choices. Nack’s study was conducted within a Western culture (United States), according to the norms and values prevailing in that country. There is thus a need to document the experiences of women who have decided to have abortions and who are thus part of the ‘bad tribe’ in Pakistan.

Previous studies have documented that the members of bad tribes are seen as deserving of stigma due to their own personal failings [31, 34–37]. Furthermore, as stated by Kumar and colleagues [32], ‘[t]he fact that so many women do have abortions, despite powerful barriers, indicates that this is contested space where agency and resistance are dynamic’ (p. 628). Our study focuses on this contested space in order to investigate the agency and experiences of women. The results can contribute to the body of literature by documenting the contested space or controversial state of women navigating between bad tribes (undergoing abortion) and good tribes (managing social status)—women exercising agency within a patriarchal society and opting for abortion. The aim of the study is to document the experiences of women who have opted for induced abortion in a society where abortion is heavily stigmatised, as well as how they navigate stigma in their daily lives.

### Research methodology

#### Research setting

Punjab province is home to more than half of Pakistan’s population. It is the most populous region on the country’s eastern side, comprising 36 districts, including Nankana Sahib. This study was conducted in a rural village in this district. The village is located off a main road and connected to other villages through a collateral road. It is religiously diverse, with two communities—Christian and Muslim—living together. In 2020, the village had 3,004 inhabitants (BHU Record Book, 2020).

The village is located in a fertile area, and it depends primarily on agriculture (both farming and livestock) for its economic subsistence. Men are also engaged in government and private employment, including transport, factory work and labour. Although women are primarily responsible for homemaking, some also perform other jobs. Christian women are more likely (as a percentage) than Muslim women are to be employed in the community. Common jobs for women include teaching, midwifery and housekeeping. The infrastructure of the village consists of cement streets, a semi-developed sewerage system, two secondary schools, one BHU^3^ (a government-based health facility), and several churches and mosques. The housing pattern is mixed.

The BHU of the village addressed in this study provides basic health facilities to the population of the village and its neighbourhoods. The BHU staff consists of one male physician, four lady health visitors (LHVs), two lady health workers (LHWs), one vaccinator and administrative staff. The staff members serve according to their scheduled hours on duty.

#### Approval

The Ethical Committee of the Faculty of Political and Social Sciences of Ghent University, Belgium, granted ethical approval for this study in 2018 (under no. 2017-37). Permission to work in the community was obtained from the responsible authorities, with the help of the Health Services Academy, Pakistan (host institution).

#### Study participants

At first, a list of target women was collected from the lady health workers (LHWs) who were active in the community [40]. Target women, older females and healthcare providers were contacted through LHWs. The first author visited them in order to make their acquaintance.

#### Informed consent

A brief overview of the research was discussed with the research participants, and their consent was obtained, either in writing (from literate participants) or in oral form (from participants with no formal education). The signed informed-consent statement clearly mentioned that the autonomy, privacy and dignity of the participants were regarded as important and that anonymous names and codes would therefore be used to protect the participants. In addition, a cover letter was prepared (by consensus of all authors) and read aloud in front of the participants before conducting the interviews and focus group discussions (FGDs).

#### Interview guide

An open-ended questionnaire guide and FGD guideline were prepared and pre-tested by the consensus of all authors. Several additions and deletions were made throughout the course of the fieldwork, as this study was part of broader research project. Initial data collection provided foundational insights into the topic. Given that the study adheres to grounded theory methodology, a second round of interviews was conducted to gather more in-depth information on abortion. Saturation in data collection was deemed to have been reached when interviews consistently yielded similar information, with no new themes emerging.

#### Data collection

This research is part of a larger study. The sample for this specific investigation was selected through purposive sampling, which made it possible to select participants who would be most relevant and who could provide the most insight into the research topic [41]. To this end, the participants selected for this study were women who had experienced abortions. Data were collected through semi-structured, in-depth, face-to-face interviews and FGDs. All interviews were conducted in a separate room in the women’s homes to avoid external pressure from their families. Similarly, the FGDs were conducted in a quiet location selected by the participants. The researcher conducted all interviews and FGDs, and a research assistant helped to take notes during the FGDs.

The fact that all interviews and FGDs were conducted by the first author (a woman of Pakistani origin) allowed the exploration of themes in a culturally appropriate manner, as the researcher could easily contact women for interviews. Moreover, as a mother of two children, the researcher could more easily address the sensitive topics of motherhood, relationships, sexuality and health matters. To avoid potential risk of discussion on religious matters, the researcher always used terms that were not in contradiction to those used in religious discussions. In addition, the researcher consistently adhered to the dress code prevailing in the village. Given the sensitivity of the topic of abortion, the researcher took care to avoid making any negative comments regarding abortion and adopted an empathic and open attitude during the interviews.

The researcher’s ability to speak both the local and national languages facilitated conversation, probing and data collection. The interviews and FGDs were conducted in either the local language (Punjabi) or the national language (Urdu), depending upon the preferences of the respondents. The interviews lasted between 45 and 60 min, with the FGDs lasting between 120 and 190 min. All interviews and FGDs were recorded, after obtaining consent from the participants.

The data for this article are based on 38 semi-structured, in-depth interviews with 24 women (pregnant—primiparous and multigravida—nursing, or having undergone induced abortion in the previous 12 months), nine healthcare providers (two traditional birth attendants (TBAs), four LHVs, two LHWs and one community midwife), and five older women. Data were also obtained from six FGDs with 12 partners, four healers and 16 older women. The characteristics of the participants are presented in Table 1.

**Table 1:** Demographic characteristics of the interviewees

Characteristics	Number
Total women	24
Christian	11
Muslim	13
**Age group (years)**	
21–25	3
26–30	9
31–40	6
40 or older	8
**Marital duration (years)**	
1–5	5
6–10	8
10–15	3
15 or more	8
**Education (women)**	
No formal education	5
Primary	6
Middle	3
Matric	8
**Husband’s occupation**	
Agriculture	9
Labour	5
Factory worker	4
Rickshaw driver	1
Other	5
**Woman’s occupation**	
Homemaker	20
Employment outside the home	4
**Number of induced abortions**	
One	12
Two	7
Three or more	5
**Remedies used for abortion**	
Home remedies	24
Self-medication	5
TBA	6
LHW	8
LHV/Gynaecologist	5

All of the women mentioned in Table 1 had used home remedies.

All interviews and FGDs were transcribed in the local/national language and then translated as a whole into English [42, 43]. The transcriptions were rechecked several times by the researcher, as well as by two language experts, one in the local language (Punjabi) and English, and another in the national language (Urdu) and English [42, 44].

### Data analysis

The data for this study were analysed according to a grounded theory approach, as outlined by Charmaz (2006) [45], who describes it as a method for discovering theory through the analysis of data. Grounded theory is an inductive approach involving several steps. The first step, open coding, consists of labelling each line of text to discover emerging information. The second step, axial coding, involves grouping similar codes together and comparing them to discover the relationships between them. In the final step, themes/categories are identified by connecting codes together to create a model of the results.

The data were arranged using the software package NVIVO 12. Before coding, the transcripts of the in-depth interviews and FGDs were read iteratively by RS. After each phase of coding, the insights that had emerged were discussed in an iterative process involving all authors. In the first round (open coding), the transcripts were coded with a focus on the following questions: (1) What is happening in the data? (2) What information is emerging? (3) What information do the data provide? For example, at this stage, the concept of motherhood norms was emerging in several transcripts: ‘mothers always take care of their children’, ‘a mother cannot harm her child’, ‘a mother’s middle name is “sacrifice”’. In the second round focused coding, similar codes were grouped together under over-arching codes. In this phase, frequent or significant initial codes were sorted to synthesise, integrate and organise a large volume of data [45]. The constant comparison of data within the categories created a ‘point of departure’ from which to organise and interpret the qualitative data. For example, codes relating to norms of motherhood were grouped under one code, with those reflecting negative ideas regarding women who undergo abortion being grouped under a separate code.

In the third and final phase, these focused codes were arranged into themes. At this stage, similarities, differences and general patterns were identified. The previous data were re-analysed whenever a new category emerged in a given interview/FGD. Advanced categories were developed during this phase, and existing ones were narrowed down. This back-and-forth process linked the emerging categories together to provide a consistent picture of the results according to dramaturgical theory. The two main components of the dramaturgical theory (frontstage and backstage) were used as sensitising concepts. The following section provides an explanation of these concepts within the theoretical framework.

### Theoretical framework

Erving Goffman’s dramaturgical theory, presented in *The Presentation of Self in Everyday Life* [46–48], explains the differences between public (frontstage) and private (backstage) behaviours. In frontstage, people act according to audience expectations, adhering to social decorum and often suppressing personal feelings [49]. Backstage, in contrast, allows individuals to act freely, without the pressures of performance, revealing a more authentic self. This theory illustrates the way people manage impressions, similar to actors preparing and rehearsing for roles in a theatre.

Frontstage regions are characterised by adherence to social scripts and decorum, where actors aim to uphold appearances through behaviours like politeness. The backstage provides a release from these expectations, allowing individuals to prepare and recuperate from the effort of maintaining frontstage roles. Goffman’s ideas have been applied to various social contexts, including social media [50], news [51], courtrooms [52], classrooms [53], hospices [54] and meat consumption in Hindus [55] and highlight the tension between individuals’ public and private selves.

One critique of Goffman’s theory is that it suggests a deceptive aspect of self-presentation in frontstage settings [56 p. 547]. While critics argue this implies inauthenticity, proponents counter [57 p. 124] that frontstage behaviours serve impression management [58] rather than manipulation. Goffman’s framework is relevant to understanding how Pakistani women, particularly in rural areas, navigate frontstage and backstage dynamics when dealing with socially stigmatised experiences like abortion. In Pakistan, abortion is widely condemned, and women often conceal it from family members to avoid social repercussions. Applying Goffman’s concept of ‘setting,’ [46, p. 22] women select specific times, places, and audiences to discuss or reveal this experience, choosing trusted inner circles as their backstage. These private spaces are not defined by physical boundaries but by the presence of trusted individuals, providing a safe space where they can express themselves more openly.

In Pakistani culture, the concepts of *muqaam* (place), *moqa* (timing), and *kon* (people/who) shape social interactions. A common saying, *gal wele di*, emphasises the importance of appropriate timing, place, and audience. This cultural framework underscores the importance of discretion in public behaviour, as personal actions are judged according to family and societal norms [59]. The study of these women’s experiences highlights the complex interplay between public expectations and private realities, with Goffman’s theory illuminating how they navigate self-presentation [60] across frontstage and backstage contexts. This theoretical framework guided the analysis by focusing on the behaviours of women within their personal circles and in public. It highlights the ways in which women present their personal experience (induced abortion) in public regions, while navigating the dynamics between the ‘frontstage’ and ‘backstage’ regions.

## Results

One core category emerging from the data concerned the fact that women who had opted for induced abortion had devoted considerable effort to avoiding stigma. This core category was derived from three inter-related categories: (i) cultural positioning of abortion and miscarriage; (ii) backstage activities; and (iii) frontstage activities and their sub-categories (see Table 2).

**Table 2:** Constructed categories based on the data collected regarding ‘avoiding stigma in cases of induced abortion’

Core category	Categories	Sub-categories
Women who opt for induced abortion and apply stigma-management strategies	Cultural position of abortion and miscarriage	Induced abortion as a cause of stigma
		Miscarriage as natural and acceptable in the community
	Backstage activities	Secret attempts to abort a pregnancy
		Active support system and its activities
	Frontstage activities	Abortion staged as miscarriage
		Abortion memories: Women coping with recollection of memories

### Induced abortion as a cause of stigma

Abortion is regarded as a violation of the norm of motherhood. A mother is perceived as a person who is always concerned with the child’s benefit. Community members believe that ‘sacrifice’ is a mother’s middle name, as she is assumed always to subjugate or sacrifice her own wishes to the benefit of her child. These points were discussed in one FGD:

*“A mother always stands in front of her child when a bad event is coming to that child; she sucks out the evil*,* but saves her child.”* (Older woman, 56 years).

*“A mother’s middle name is ‘sacrifice’.”* (Older woman who had experienced miscarriage [years ago], 51 years)

Members of the community thus believe that a mother is incapable of harming her child. A mother’s love is exceptional, and other relationships cannot emulate it. In the Pakistani tradition, a mother’s highest priority is to love and sacrifice for her child. Women who go against this norm are therefore not considered good mothers, and they are subject to resistance and stigma from the community. This point is addressed in the following quotations:

*“Women who have killed their child with their own hands…are killers.”* (Woman who had experienced miscarriage [years ago], 71 years)

*“These women are like snakes…you know…a snake eats its children by itself…so these women are like sapni* [snakes]”. (Older woman who had experienced miscarriage [years ago], 54 years)

As demonstrated by the excerpts presented above, women who have undergone induced abortion are regarded as ‘non-mothers’, and community members are likely to relate them to animals that are capable of harming their own children. Respondents assigned the labels of ‘killer’ and ‘murderer’ to women who would end the life of a child in the womb. Furthermore, community members see induced abortion as an act against the will of God, and women who do so are challenging his authority. Abortion is thus regarded as running counter to the norm of motherhood and the image of a ‘good’ mother. For these reasons, women who opt for abortion are subject to stigma.

Community members use the following statement to refer to induced abortion: *is ne bacha zaya kerwa diya he*—‘She gets her child wasted deliberately’. This statement makes a direct reference to a woman’s bad intentions towards her unborn child. Abortion is equated with wilfully harming a child, and the woman is regarded as a culprit.

Another statement used to refer to abortion is as follows: *ye to apni aulad ki saggi nahi he*—‘She is not sincere with her children’. This statement also bears an inherent stigma and creates a negative image of the woman.

### Miscarriage as natural and acceptable in the community

This theme concerns the fact that miscarriage is acceptable within the community. As acknowledged by respondents, not all pregnancies are successful. Community members tend to attribute miscarriage primarily to the will of God. It was discussed as follows in one of the FGDs with older women:

*“God decided for the life of each human being…he grants the life…For some* [people], *he grants many years of life…for some [people]*,* he grants only a few years*,* months or days.”* (64 years).

*“Yeah…some children are not born alive…a few just end in the womb.”* (52 years).

The excerpts presented above reflect a belief that life and death are under control of God, who is the ultimate authority. Humans can therefore not question Him when He ends his life in the womb. Community members noted that miscarriages can occur due to supernatural interventions (e.g. sorcery, possession and the evil eye). This is illustrated in the following quotations:

*“Pregnancy is a critical phase…the evil eye and sorcery can harm the child in the mother’s belly* [*womb*].*”* (Woman who had experienced miscarriage, 39 years).

*“Parchawan* [evil shadow] *of a woman with athra* [a maternal disease] *can cause miscarriage.”* (Woman who had experienced stillbirth, 26 years).

Respondents stated that the elements mentioned in the excerpts can cause the loss of a pregnancy. Although a woman can exercise caution, these circumstances are beyond her control. A pregnant woman is thus always expected to take care of herself for the sake of her foetus, but mishaps are bound to occur. This is reflected in the following quotations:

*“My three pregnancies went well*,* without any issues…but my fourth pregnancy ended in abortion* [miscarriage].*”* (Woman who had experienced miscarriage, 29 years).

*“I had an abortion* [miscarriage] *in the fifth month of pregnancy…I was busy doing household chores for the whole day…in the night I had an abortion* [miscarriage]*…After that*,* my six pregnancies have been successful.”* (Woman who had experienced miscarriage, 36 years).

*“A tree has many fruits in the season…some fruits ripen…some fruits leave the plant before ripening and some fruits just do not grow and finish in earlier days…a woman’s womb is also like a plant* [where not all fruits grow to maturity].*”* (Older woman, 58 years).

As illustrated by these excerpts, miscarriage is accepted within the community. Respondents expressed a belief that not all pregnancies can be successful. Mishaps can occur. Such losses are thus considered normal and part of the reproductive trajectory. These losses are bearable, and women who experience them are not regarded as culprits. Many of the community members used the statement *bacha zaya ho gaya he*—‘A kid has been wasted’—to describe miscarriage. This statement does not imply that the woman is a culprit. It is purely an expression of loss. Another statement used during a discussion was *Is bechari ka bacha zaya ho gaya he*—‘She [poor girl] has wasted [lost] her child’. This statement expresses the helplessness of a woman with regard to miscarriage. The word *bechari* ‘poor girl’ is used to show sympathy for her helplessness. In most cases, conversations about miscarriage are initiated with verbal expressions (e.g. *o-hoo*,* haan-haa*,* haie haiehay*,* cheh cheh cheh*,* ooi*) to express sympathy.

The acceptance of miscarriage within the community provides room for women who have opted for induced abortion to stage the abortion as miscarriage in order to avoid negative impressions in the community. The following themes express how these women create the scenario to stage induced abortion as miscarriage.

## Secret attempts to abort a pregnancy

This theme focuses on the attempts of women to abort their pregnancies in secret. Community members expressed that women usually resort to some traditional method when opting to terminate a pregnancy, as illustrated by the following quotations:

*“I drank a decoction of herbs…I was bleeding on the third day.”* (Woman who had experienced abortion, 39 years).

*“I carried heavy weight…I was washing blankets and remained busy with that for the whole day.”* (Woman who had experienced abortion, 29 years).

*“I jumped several times daily from different home items*,* such as stairs*,* a cot and a sofa.”* (Woman who had experienced abortion, 26 years).

Amongst the traditional methods that respondents reported having used to abort their pregnancies were foods (e.g. raisins, dates) and other edible items that are considered to have hot properties that can cause bleeding. Similarly, jumping, skipping rope, carrying heavy objects and hurriedly going up and down stairs are perceived as capable of causing miscarriage. Women thus resort to these methods to abort their pregnancies in secret, out of sight of their families. These methods work in some, but not all cases. For this reason, some women use medicines to induce abortion.

*“I went to a gynaecologist…and asked for medicine…she scolded me* [for wanting to abort the pregnancy], *but gave me medicines.”* (Woman who had experienced abortion, 38 years).

As mentioned above, the respondents had performed all of these attempts in secret, and their families had no information about them. Only a few people (e.g. a sister, a close friend) knew about the decision to abort the pregnancy.

### Abortion staged as miscarriage

Many of the women in this study staged their abortions as miscarriages in front of their families and the community. They did not disclose the reality of the situation. Family members received news of physical problems (e.g. backache, bleeding), interpreted these signs as alarming and came forward to help the woman.

*“I was bleeding heavily…my mother-in-law called the TBA for a checkup… The TBA said that I had experienced a spontaneous abortion* [miscarriage].*”* (Woman who had experienced abortion, 51 years).

*“When my family took me to doctor…they said that I had slipped while working at home…that I was bleeding.”* (Woman who had experienced abortion, 27 years).

Respondents reported having had signs of miscarriage (e.g. bleeding, backache) after using decoctions, herbs or medicines. When these signs occur, the backstage region shifted into the frontstage region. Women involved their family members (e.g. parents or in-laws) in the story of the abortion (staged as a miscarriage). The family members then came forward and took the initiative to cope with the unsuccessful pregnancy. Women expressed that they had kept these secrets for their whole lives, as it would be disastrous for them if their families—especially their husbands—were to receive information regarding the abortion.

Women performed the initial steps to induce abortion in secret. After a few days, the situation manifested as miscarriage due to medical need. In some cases, however, the abortion occurred without any assistance or medical aid, and the family was not aware of it at all. These abortions remained a secret existing only in the memory of the woman involved.

*“I have had three* [induced] *abortions in total*,* but my husband and family only knew about one…they had no idea about the other two* [induced] *abortions. My sister and I know about them.”* (Woman who had experienced abortion, 61 years).

As illustrated by the excerpt above, women often exercise their agency in secret, after having secured their status within the family. These women make decisions by themselves, without involving their husbands. Abortions can also reflect women’s agency, in which they involve their husbands when it becomes essential. In most cases, women keep their husbands as far away from the story as possible.

### Active support system in the frontstage and backstage regions

This theme concerns the support systems that function in the backstage region to help women in the process of induced abortion. In most cases, these systems consist of a few family members and friends. This is illustrated by the following quotations:

*“My sister-in-law helped me. I went to my mother’s home. She* [my sister-in-law] *accompanied me to visit a doctor for a prescription. She told my mother that she was going to visit the doctor due to a headache…I was going to go with her.”* (Woman who had experienced abortion, 27 years).

*“My friend helped me…Her in-laws went to attend a funeral in another city…She was alone at home during the day…We called the TBA from her home to discuss the situation.”* (Woman who had experienced abortion, 35 years).

In this way, family and friends helped women to arrange to meet with healthcare providers. They staged matters under their own names in order to provide safety for the women. The support systems also helped to provide information for handling the situation. As described by one respondent:

*“My sister had experienced a spontaneous abortion* [miscarriage]*…she told me what can happen in that situation…She also told me I should look like a sick person who is not feeling well. She also told me I should not visit a doctor or look lazy.”* (Older woman who had experienced abortion [years ago], 62 years)

In the backstage region, members of the support system provide information about frontstage expressions and performance in front of the audience or family members who are not part of the backstage region (in this case). In some cases, women in the backstage regions talk about the story. They also provide safety to women in the frontstage regions and try to avoid any situations that could disclose the true situation. One woman stated:

*“My mother-in-law carried me to the BHU…one of my friends was with me…She was discussing with the LHV and did not allow my mother-in-law to contact the LHV…the LHV knew the reason.”* (Woman who had experienced abortion, 38 years).

As described by respondents, the support system (always women) helps women to control situations in the frontstage region. Backstage participants try to manage the situation and hide the reality, avoiding any circumstances that could break news of the secret events. In the present study, the backstage participants had helped women at crucial times (e.g. when the woman was in hospital or not in a good physical condition due to the abortion). When the medical staff were aware of the reason, members of the support system controlled the situation by talking to members of the medical staff and the woman’s family. They also helped women later, whenever the same event was discussed. As illustrated by one respondent:

*“My friends and I were discussing my abortion a few days after the event…My mother-in-law was not at home…We were discussing the story*,* and I was relaxed…Suddenly…my mother-in-law entered the home…My friend became quiet…I started sobbing to control the situation.”* (Woman who had experienced abortion, 42 years).

As illustrated by this theme, support systems are always based in the backstage region. These participants can be relatives (e.g. sister, sister-in-law, mother, mother-in-law) or from outside of the family (e.g. friends, relatives and neighbours). The backstage and frontstage regions are thus defined in terms of the presence of an audience, and not in terms of physical boundaries. For example, the home does not constitute a physical boundary in this case. Backstage helpers could possibly be from outside the woman’s home. In some cases, the entrance of one or more individuals into the backstage region transformed it into frontstage, and the entire scenario of the conversation changed. In such situations, all backstage participants shifted to perform in the frontstage region. The backstage region is secretive, and people help each other to keep the secret.

### Abortion memories: women coping with recollections of abortion

The expressions and feelings that women express when recalling their abortions differ according to space. For example, in front of the community (frontstage), such recollections are always discussed in terms of sadness and helplessness. One respondent recalled:

*“My sister-in-law experienced spontaneous abortions* [miscarriages] *two times…it’s the will of God.”* (Woman with four children, 49 years).

*“It depends upon the will of God how much life he has written for a child.”* (Woman who had experienced an abortion, 56 years).

In most cases, the women became sad during public (frontstage) discussions regarding their induced abortions. Some even began to tear up when talking about these events.

As shared by our respondents, community discussions that relate miscarriage and abortion to God can become a point of tension, as they have violated the rule. In front of others, they may weep and, whilst others attribute their tears to sadness, they are actually weeping for having committed a sin. Some women referred to a pain deep in their hearts, and they remained tense and emotionally burdened when recalling their abortions in private.

*“I felt guilty when my son was ill…I thought it was punishment.”* (Woman who had experienced an abortion, 38 years).

*“I ask forgiveness from God…for my sin.”* (Woman who had experienced an abortion, 48 years).

As illustrated by this excerpt, some women felt so burdened that they would sometimes interpret negative events (e.g. the illness of a living child) as punishment for having had an induced abortion. Some were afraid that God might take back their living children or create other problems (e.g. loss of the husband’s job, illness of a family member). Such fears led some women to secretly ask forgiveness from God or to weep in isolation when reflecting on what they had done in the past.

In contrast, some women expressed the belief that, although they had committed a sin, it was justified by the fact that they had no other options. Furthermore, they were convinced that God would not be angry, as he understood their situations better than they did themselves. This appears to be a means by which the women tried to justify their actions to themselves and to eliminate the burden posed by their abortions. As expressed by one respondent:

*“I am okay with what I have done…It is a sin*,* but what I can do now?”* (Woman who had experienced an abortion, 61 years).

In other cases, women reported that they did not feel any guilt, but that they felt obliged to look sad in front of others. Because members of the community—especially women—perceive miscarriage as a loss to the family, a woman is supposed to recall this loss by weeping, looking sad and uttering words that reflect helplessness in light of the lost pregnancy. In these cases, the women adopted or staged their behaviour in an acceptable manner in order to maintain decorum. This is illustrated by the following quotations:

*“Every time my grandmother-in-law recalls my miscarriage* [induced abortion]*…she hugs me and weeps…I feel* [myself] *in an awkward position…because I am supposed to look sad…I try hard to look sad and weep.”* (Woman who had experienced abortion, 34 years).

*“It is really difficult to face the situation when my mother suddenly starts talking about my miscarriage* [induced abortion].*”* (Woman who had experienced abortion, 28 years).

In the first of these two excerpts, an older woman expressed sympathy and tried to reduce the younger woman’s pain due to miscarriage. Instead of being comforting, however, these sympathetic moments became a burden for the women. They disliked these sympathetic moments and became tired of having to act accordingly or appropriately again and again. Such memories are personal and private, and women do not share them with their families or other public figures. These acts are performed in private (backstage).

This theme reflects the dual nature of memories regarding induced abortion. In the frontstage region, women are likely to recall their abortions by looking sad and portraying the experience as a loss that happened naturally. In contrast, in the backstage region, such memories are expressed in different ways. Some women may feel a psychological burden, which they attempt to alleviate by asking for forgiveness in secret and finding justifications for their actions.

## Discussion

In our study, we apply Goffman’s dramaturgical (frontstage/backstage) theory to the context of induced abortion. This theory concerns differences in the frontstage and backstage behaviours of the same people. This study explores the behaviours of women in both spaces with regard to the experience of induced abortion, in which community members break prevailing social norms and taboos according to their own agency.

In addition to confirming current literature, this study has yielded a number of new findings. The results confirm that, in the Pakistani society, motherhood is the most important norm for women, and women who are viewed as going against this norm are stigmatised. Our study is the first to apply Goffman’s dramaturgical theory to the topic of abortion within the context of Pakistani culture. This theory has been criticised for not adequately addressing cultural diversity and for being applied universally, across all cultures [58, p. 290]. Similarly, while many key concepts have been employed in previous studies, significant differences can emerge when examining behaviours within specific cultural contexts. For example, this study demonstrates how the use of space shifts with the presence of different people. Physical boundaries do not function as clear distinctions between ‘backstage’ and ‘frontstage’. For example, when only members of the inner circle are present, the space—whether at home or elsewhere—functions as a backstage region. Upon the entrance of an outsider, however, this backstage region is transformed into a frontstage region. This dynamic was evident in several instances recounted within the study, as when women discussed their abortions openly with friends but immediately begin to sob when someone outside their inner circle entered the space. In addition, members of the inner circle and those within the backstage environment often assisted women in their attempts to manage the situation during critical moments.

Similarly, the study highlights various backstage activities, including the use of language, behaviours and abrupt changes, as well as the adoption of behaviour according to who is listening. This was evidenced by situations in which women suddenly adopted frontstage behaviours that are in accordance with broader group norms, even though their backstage behaviours are likely to deviate from these norms. For example, a woman might express negative opinions about induced abortion in public, even after having undergone the procedure in secret [25].

This study also reveals differences in traditional norms relating to the family. Given the patriarchal character of Pakistani society, most decisions are made by men. The results of this study nevertheless indicate that women also make decisions in the backstage regions. The findings thus demonstrate that women sometimes do violate the power of men, albeit secretly. The study highlights the significance of group norms within a culture and emphasises the crucial role that friends play in maintaining secrecy. This underscores the allocentric nature of friendships in Pakistan and other collectivist cultures [61]. More specifically, induced abortion constitutes a deviation from socially important or sacred norms that affect both the culprit (i.e. the woman who undergoes the procedure) and the members of her in-group [62], who often constitute the frontstage audience or participants.

Social norms are important in any society, including Pakistan. For this reason, people always try to act in accordance with the norms. They are also concerned with regard to their social status in the community. Given the importance of social status to maintaining respect within society, women who opt for abortion are likely to hide such information from their families and relatives. As illustrated by the women participating in this study, they may stage abortion as miscarriage, which is regarded as a natural loss and is thus accepted within the community and generates sympathy and less stigma. Because induced abortion is not accepted in Pakistani society, women who opt for abortion are regarded as non-women and non-mothers. They are seen as killers and murderers [63, 64]. In some cases, community members may even equate them with dangerous animals that eat their own offspring (e.g. snakes). They are likely to be judged negatively by their families and other members of the community [65–68], thereby resulting in the loss of social status. Similarly, the decision to share information with an audience depends largely on the nature of that audience. The women themselves are thus likely to be the only ones who know exactly how many pregnancies and abortions they have had [69]. As illustrated by the experiences shared by the women participating in our study, pregnancies that are terminated in secret are often staged as miscarriages. This indicates that women selectively conceal and disclose information regarding their pregnancies and abortions [70, 71], depending on the audience. In frontstage spaces, women recall their abortions as a natural loss and behave in a socially accepted manner (e.g. by weeping, sobbing and expressing words of sorrow at the loss of the foetus). In the backstage regions, women exhibit different behaviours. Some women are satisfied with the decision to undergo abortion, others regard the abortions as a sin and ask forgiveness, and yet others are likely to interpret adverse life events (e.g. the illness of a child or economic setbacks) as punishment for having had an abortion. The feelings that women have regarding abortion thus vary substantially [70, 72]. As demonstrated by our study, the ways in which women recall and express their feelings about abortions differ greatly between public (frontstage) and private (backstage) spaces.

Our results highlight strong behavioural discrepancies in cultures where social norms are strong and where individuals have less say in expressing their wishes and desires. In such cultures, individuals often use backstage spaces to fulfil their wishes. For example, a study conducted in India reports similar results for Hindu individuals with regard to meat consumption. The need for such differences in behaviour is especially likely to arise in cultures where people are pressured to follow strong group norms [73, 74], and where those who disobey or deviate from these norms are perceived as sinners. In some cases, non-conformists are designated as abject beings, who are then subject to stigma and blame, as discussed by Scambler (2019) [75].

Given that abortion is allowed legally and religiously throughout the first trimester, it is essential for abortion to be destigmatised. Several stakeholders can play role in this regard, including policymakers, healthcare providers and community members. According to the literature on destigmatisation, powerful individuals can play a fruitful role by initiating and carrying out changes in structures [76]. Within this context, policymakers could initiate campaigns regarding destigmatisation for women undergoing induced abortion through the healthcare system and social media.

As highlighted by Samad et al., social media campaigns can play a crucial role in mitigating societal stigma [77]. Social media campaigns raise awareness regarding legal and religious permissions/possibilities for abortion. Furthermore, campaigns regarding use of contraceptives could reduce the number of unintended pregnancies, and they could thus ultimately reduce the abortion rate. In Pakistan, positive strides have already been made with a successful social media campaign addressing birth control through contraceptives [77], thereby underscoring the potential impact of such efforts.

Other influential figures could also help by sharing information regarding abortion at the religious, legal or community level. As identified in this study, these figures could include religious healers, lady health workers (LHWs) and traditional birth attendants (TBAs). These individuals hold sway within the community, making their involvement pivotal for initiating positive change. The influence and credibility wielded by religious leaders and TBAs make them particularly well suited to address the community effectively. Given that LHWs provide healthcare services directly to women in their homes, their participation adds a unique dimension to the destigmatisation strategy.

The national healthcare department should provide training to sensitise staff members with regard to abortion. They should also restrict untrained staff (e.g. LHWs and TBAs) from performing abortion procedures. The healthcare department should also take the initiative to inform people and publicise the risks associated with clandestine abortions, in addition to taking appropriate measures to introduce methods of family planning that could prevent/avoid unintended pregnancies.

Community members can play a crucial role in the destigmatisation of abortion. Families should educate young girls, thereby helping to change attitudes towards women (who are stigmatised). Furthermore, young women tend to respond positively to women who have been subjected to societal stigma. Community members should learn to adopt an independent stance regarding rules (either religious or legal) concerning matters such as abortion.

## Conclusion

Our study demonstrates that social pressure and the desire to avoid stigma can lead women to undergo abortion in secret. In Pakistan, clandestine abortions are the cause of substantial levels of maternal mortality and morbidity, and many women experience a wide range of physical issues, including infection and haemorrhage. It is therefore important to discuss this topic at the societal level. The results of this study should encourage influential figures to come forward and play a role in addressing the root cause of the stigma surrounding abortion. For example, the relevant department should work to decrease the incidence of unintended pregnancies. Similarly, the health department should develop standards for professional practice based on clear information regarding abortions laws (both legal and religious).

At the community level, debate and discussion could make a positive contribution to the destigmatisation of abortion. At present, such discussion and communication are restricted by taboos surrounding this topic. Initiating the discussion and reducing the gap in communication could lead to the dissemination of useful information that could help to avoid harmful results.

One important strength of this study is that it considers the experiences of women with regard to abortion by examining the ways in which they manage social status and psychological burden within a community in which abortion is stigmatised. The results of our study further reveal that women risk their physical and psychological health by undergoing induced abortion. It also points out that, although women do exercise agency to taking such decisions, they are likely to feel guilt throughout the rest of their lives.

Our study is also subject to a number of limitations. For example, in terms of grounded theory, the researcher’s positionality as a woman might have had some influence on the outcomes of the study [78]. To minimise the possibility of such bias, perspectives were gathered from multiple researchers with regard to the data [79]. The triangulation of data sources also enhanced the collection of diverse viewpoints [80].

The body of literature addressing the issue of induced abortion is growing. Based on the results of this study, future research could explore health problems that occur due to induced abortion and how these problems affect the lives of women and their families.

## Electronic supplementary material

Below is the link to the electronic supplementary material.

Supplementary Material 1

## Data Availability

The datasets generated and/or analysed during the current study are not publicly available due [We are sorry that we could not share the raw data because it includes the original information/ identity markers of the participants. The participants do/were not agree to share the raw data outside of our team. We could not share the data publicly for the participants’ privacy] but are available from the corresponding author on reasonable request.

## Footnotes

1 In a patriarchal society, males hold all forms of power (e.g. decision-making).

2 Pronatalism is a norm prescribing that every couple should have at least one biological child.

3 The public healthcare sector in Pakistan is a three-layered structure [38]. Primary healthcare (PHC) comprises basic health units (BHUs) and rural health centres (RHCs), which serve rural areas. The BHUs cover around 25,000 inhabitants, and the large RHCs cover 1,00,000 inhabitants. The BHUs and RHCs are responsible for providing preventive, curative and referral services. Their services also include maternal and child healthcare (MCH). Secondary healthcare comprises *tehsil* headquarter hospitals (THQs) and district headquarter hospitals (DHQs), which serve cities (*tehsils* and districts). The tertiary healthcare sector comprises health institutes, which serve metropolitan cities. The Pakistani healthcare system consists of both private and public providers. Private healthcare services include an array of folk healers, religious/spiritual healers and traditional healers (e.g. traditional birth attendants, bone setters, massage service providers), as well as herbalists, nurses, laboratory technicians and certified practitioners [39].
