# Supplementary material for: ‘It’s a secret in my life’: frontstage and backstage experiences of women undergoing induced abortions in a rural community of Pakistan
Source: BMC Womens Health. 2024 Dec 31;24:676. doi: 10.1186/s12905-024-03482-5 (PMC11687180; doi:10.1186/s12905-024-03482-5)
Supplement: Supplementary file 1 — Supplementary Material 1 [file 12905_2024_3482_MOESM1_ESM.docx]

**Interview guide**

This interview guide is showing the questions that are relevant to this study.

**Interview guide (General for 1^st^ round)**

1. Do you have any children?
2. How many times have you been pregnant?
3. Have you ever experienced a miscarriage or abortion?
   (A miscarriage is natural, and an abortion is induced.)
4. How many miscarriages or abortions have you experienced?
5. At the time of your miscarriage or abortion:
   - Age of the woman
   - Month of pregnancy
   - Number of children (she already has)

**Interview guide 2^nd^ round**

Here is a revised version of your list with improved grammar:

1. How or why did you decide to have an abortion?
2. When did you decide to have an abortion?
3. Did anyone know about your decision?
4. Did anyone help you make this decision?
5. Who helped you?
6. Did anyone assist you practically with the abortion (e.g., by suggesting something)?
7. Was it a friend, sister, sister-in-law, mother, or mother-in-law?
8. Did your husband, in-laws, or parents know about it?
9. What actions did you take?
10. Did you use any medicine, food, or something else?
11. What happened afterward?
12. Which remedy or medicine worked for you?
13. What happened after consuming this food?
14. How many days did it take?
15. Did you visit any doctor or traditional birth attendant (TBA)?
16. Who accompanied you to the healthcare provider?
17. What happened at the healthcare facility (hospital, TBA home, LHV clinic, etc.)?
18. How did you feel physically after the abortion?
19. How did you manage your children during this time?
20. Did you have any support person?
21. How did you feel about your decision emotionally?

**Interview guide 3^rd^ round**

1. How do you remember your abortion?
2. How do you feel about the abortion in your daily life? Do you feel happy, sad, or indifferent?
3. Are you happy with your decision?
4. What do you do when this memory comes to your mind? Do you react alone or in front of people?
5. Have you informed your husband or others about the abortion? Why or why not?
6. How did you handle the situation in the hospital? (Did the doctor tell your husband about the abortion?)
7. How many people know about your abortion?
8. What do you do when people discuss your abortion?
9. How do you respond?
10. Do you feel any fear from a friend who knows the truth?
11. Do you discuss this memory with your friends? (Do all your friends know the story?)
12. How do your friends feel about it, or how do you discuss the matter with them?
13. What do you think about people who view abortion negatively?
14. What are your thoughts on abortion? Should other women consider it?
15. What is your opinion regarding abortion, whether in private or public discussions?
16. Do you feel any changes in your life after having this abortion?
